# Reducing Intrusive Trauma Memories Using a Brief Mental Imagery Competing Task Intervention: Case Series of Trauma-Exposed Women in Iceland

**DOI:** 10.2196/72748

**Published:** 2026-05-15

**Authors:** Kristjana Thorarinsdottir, Emily A Holmes, Johann Hardarson, Freyja Agustsdottir, Marie Kanstrup, Laura Singh, Arna Hauksdottir, Thorhildur Halldorsdottir, Berglind Gudmundsdottir, Edda Bjork Thordardottir, Unnur Valdimarsdottir, Andri Bjornsson

**Affiliations:** 1 Department of Psychology, University of Iceland, Saemundargata 12, Reykjavik, 102, Iceland, 354 5254240, 354 5254240; 2 Department of Women’s and Children’s Health, Uppsala University, Uppsala, Sweden; 3 Uppsala University, Uppsala, Sweden; 4 Department of Psychology, Uppsala University, Uppsala, Sweden; 5 Swedish Collegium for Advanced Study, Uppsala, Sweden; 6 Center of Public Health Sciences, University of Iceland, Reykjavik, Iceland; 7 Department of Psychology, Reykjavík University, Reykjavik, Iceland; 8 Department of Medicine, University of Iceland, Reykjavik, Capital Region, Iceland; 9 Landspitali—University Hospital, Reykjavik, Iceland; 10 Department of Epidemiology, Harvard TH Chan School of Public Health, Boston, MA, United States; 11 Unit of Integrative Epidemiology, Department of Environmental Medicine, Karolinska Institutet, Stockholm, Sweden

**Keywords:** trauma, intrusive memories, posttraumatic stress disorder, imagery competing task, visuospatial task, Tetris gameplay, mental imagery, case series

## Abstract

**Background:**

There is a need for scalable and simple interventions for trauma-exposed people. In this case series, we built on our previous case study and case series findings and further explored the use and potential effectiveness of a brief novel intervention to reduce the number of past intrusive memories of trauma. The imagery competing task intervention consists of a memory reminder and the visuospatial task Tetris played with mental rotation, targeting 1 intrusive memory at a time. Here, we test remote delivery of the intervention, including guidance from researchers without specialist mental health training, in a sample of women in Iceland with current intrusive memories from trauma.

**Objective:**

In a case series of trauma-exposed women, we aimed to explore whether this brief novel intervention reduces the number of established intrusive memories (primary outcome) and improves general functioning and symptom reduction in posttraumatic stress, depression, and anxiety (secondary outcomes). The acceptability of the intervention along with adaptations, that is, delivery by psychology students without specialist mental health training and digital delivery, was explored.

**Methods:**

Participants (N=8) monitored the number of intrusive memories from an index trauma (occurring 3‐16 years previously) in a daily diary at baseline, during the intervention, and postintervention at 1-month and 3-month follow-ups. The intervention was delivered digitally with guidance from clinical psychologists or psychology students. A repeated AB design was used (“A”: preintervention baseline, “B”: intervention phase). Intrusions were targeted one by one, creating repetitions of an AB design (ie, length of baseline “A” and intervention “B” varied for each memory).

**Results:**

The number of intrusive memories reduced for all participants from the baseline phase compared with the intervention phase, although the reduction was minimal for 2 participants (6.3%‐93%). The number of intrusive memories continued to reduce for 6 out of 8 participants (58%‐100% reduction at 1-month follow-up; 72%‐100% reduction at 3-month follow-up). Symptoms of posttraumatic stress, depression, and anxiety were reduced for most participants postintervention and continued to decrease during the follow-up periods. Functioning was improved for 7 of the 8 participants from baseline to postintervention and continued to improve at the follow-up assessments for 3 participants. The intervention delivered digitally and partly by students was perceived to be an acceptable way to reduce the frequency of intrusive memories by all participants (mean rating 9.5 out of 10).

**Conclusions:**

Data from this case series of traumatized women provide preliminary evidence for the effectiveness of this novel brief intervention in reducing intrusive memories of trauma occurring several years ago and in improving functioning and reducing core symptom burden. This study will inform a randomized controlled trial of this novel intervention, which may have considerable implications for large-scale clinical management of traumatized populations.

## Introduction

More than 70% of people experience traumatic events (eg, interpersonal violence, accidents, or disasters) at some point in their lives [1]. The lifetime prevalence for posttraumatic stress disorder (PTSD) has been estimated as 5.6% among people who have been exposed to trauma [2]. PTSD, even when symptoms are subthreshold, is associated with substantial distress, functional impairment, and comorbidities, such as depression and anxiety [3-5]. Given the scope of this detrimental health outcome, there is a need for scalable and simple interventions for trauma-exposed people.

A core clinical feature of PTSD is intrusive memories of trauma. They are often visual, for example, pictures or short clips that replay a distressing moment from the trauma. They can disrupt daily functioning and cause significant distress by evoking the same emotional reaction that was experienced during the traumatic event [6]. According to network studies [7], intrusive memories activate the other 3 PTSD symptoms clusters (avoidance, alterations in cognitions and mood, and hyperarousal), making them a suitable treatment target [8]. This single symptom focus may allow a posttrauma intervention to be simpler and easier to scale.

Existing evidence-based treatment options for PTSD [9,10], while clearly important, also carry some limitations. These treatments require the patients to recall and describe the traumatic event in detail, which many are reluctant to do [11]. In addition, dropout rates from clinical trials focusing on PTSD treatments are high, up to 48% (on average 18%) and are thought to be even higher in general treatment settings [12-14]. Furthermore, there are not enough therapists who are trained in delivering evidence-based PTSD treatment [11], and current treatment options are time-consuming and costly [11]. With these challenges in mind, a simple and low-intensity intervention has recently been developed, aimed to reduce 1 key symptom of PTSD, that is, intrusive trauma memories [15] rather than the full diagnosis of PTSD [16]. The intervention consists of a brief memory reminder for a specific intrusive memory, followed by 20-minute Tetris gameplay with mental rotation (ie, actively rotating the blocks in one’s mind while playing the game; for further details, see Holmes et al, Chapter 11 [17]).

Recent clinical trials have yielded promising results when the intervention is used to target memories of very recent trauma [18-20]. In several case studies and case series, the intervention has also been investigated with a focus on older intrusive memories of trauma with promising results [21-27]. These studies involved both in-person and remote delivery of the intervention with sessions being guided by a clinical psychologist or a researcher.

In a recent case study [22], we adapted the intervention for Icelandic women who experience intrusive memories of trauma. In the initial case study, the participant (n=1) was a woman in her fifties with 4 intrusive memories of a single traumatic event from her childhood. She reported a total of 12.6 intrusive memories per week at baseline, which reduced to 6.1 per week during the intervention phase. Notably, her intrusive memories continued to decrease to 1.0 per week at the 3-month follow-up. The participant’s general functioning, along with symptoms of PTSD, depression, and anxiety, improved postintervention and continued to improve at the follow-up. She found the intervention to be an acceptable way to reduce the frequency of intrusive memories [22].

Following the case study, we investigated the effects of the intervention in an exploratory case series (n=3 [23]). Given restrictions that came along with the COVID-19 pandemic (eg, isolation) [28], we also explored the feasibility of delivering the intervention remotely, using videoconferencing. The total number of intrusive memories for the first participant went from 38.6 per week at baseline to 18.0 per week during the intervention phase, for the second participant from 10.8 per week at baseline to 4.7 per week during the intervention phase, and for the third participant from 33.7 per week at baseline to 20.7 per week during the intervention phase. The frequency of intrusive memories also continued to decrease at 1-month follow-up for all 3 participants, indicating that the intervention may have longer-term effects. As in the study by Kessler et al [26], we hoped to compare targeted relative to nontargeted memories; however, the number of nontargeted memories per week was too low to make that comparison. In addition to the reduced total number of intrusive memories, PTSD symptoms were reduced for all 3 participants. Furthermore, depression and anxiety symptoms were reduced for 2 out of 3 participants postintervention and continued to reduce at follow-up. Two out of 3 participants rated the intervention to be an acceptable way to target intrusive memories of trauma. Remote delivery—here using videoconferencing with the researcher—was achieved and appeared acceptable, although the feasibility of remote delivery needs to be further explored.

In the current case series, we build on these 2 previous studies [22,23], investigating the effects of the intervention (up to 6 sessions) delivered remotely in a larger case series sample of women (n=8) experiencing established intrusive memories of older trauma occurring some years before (3‐16 years). In this study, a novel aspect was also that nonspecialists (psychology students without a professional mental health qualification) delivered the intervention remotely after training in how to use the intervention.

We predicted that participants would report fewer intrusive memories of trauma (primary outcome) during the intervention phase (maximum 5 weeks) than in the preceding baseline phase (1 week), and that the reduction would be maintained at 1-month and 3-month follow-ups. We predicted that the number of targeted intrusive memories would decrease relative to nontargeted memories. Furthermore, we explored whether having fewer intrusive memories would be associated with improvements in general functioning and reductions in symptoms of PTSD, depression, and anxiety (secondary outcomes). In addition, we explored the feasibility and acceptability of digital and nonspecialist delivery of the intervention.

## Methods

### Participants

As in our previous studies [22,23], participants were recruited from a substudy of the Stress and Gene Analysis (SAGA) cohort, which is a population-based longitudinal cohort study of Icelandic women [29,30]. The substudy compared 2 samples of women from the SAGA cohort study, women with a probable diagnosis of PTSD (ie, having a score of 33 or higher on the Posttraumatic Stress Disorder Checklist [PCL-5]) and women not likely to have a diagnosis of PTSD (ie, scores on the PCL-5 in the lowest one-fifth of the distribution), using clinical interviews. Women who took part in the substudy were assessed for eligibility for this study using 2 semistructured diagnostic interviews (ie, the Mini International Neuropsychiatric Interview [MINI] and the Clinician-Administered PTSD Scale for DSM-5 [CAPS-5]) to determine the presence of mental disorders and to assess exclusion criteria in this study. Women from the substudy who provided consent to be contacted regarding future studies were assessed for inclusion into this case series.

Screening included a short description of intrusive memories (as involuntary and distressing memories that include sensory impressions such as sight, sound, etc, that pop into mind often in the form of pictures or short film clips). The presence of the symptom was then assessed.

In this study, 53 women from the substudy who provided consent to be contacted for further research were assessed for inclusion (see CONSORT [Consolidated Standards of Reporting Trials] diagram). Inclusion criteria were (1) having experienced at least 1 Criterion A trauma according to the *Diagnostic and Statistical Manual of Mental Disorders, Fifth Edition* (*DSM-5*) [31], (2) having at least 1 intrusive memory that occurs at least 3 times per week in the past 4 weeks, (3) being able to attend 3‐8 sessions with the researcher, (4) being willing to monitor intrusive memories in daily life, (5) having access to a smartphone, and (6) being able to speak Icelandic and read study materials in Icelandic. Exclusion criteria were (1) current psychotic disorder, (2) current manic episode, and (3) being acutely suicidal.

Ten participants met the inclusion criteria and were initially recruited, ranging in age from 22 to 65 years (mean 43.2, SD 15.67 years). Participants will hereafter be referred to as P1, P2, P3, and so on. Two participants (P6 and P8) did not complete the intervention sessions due to personal reasons. P6 did not complete the baseline session, and no data were therefore collected for P6. P8 completed the baseline session and 1 intervention session. P8 found the timing of study participation not suitable due to changes in her personal life. Hence, P6 and P8 were excluded from the data analysis. Eight women completed the study (see Table 1 for demographics, traumatic event details, and clinical diagnoses for the final sample). Time since the traumatic event took place that participants were experiencing intrusive memories from varied from 3 to 16 years (mean 7.25, SD 6.60 years).

**Table 1.** Participant demographics, traumatic event details, and clinical diagnoses.

Participant	Age (years)	Traumatic event	Different intrusive memories, n	Clinical diagnoses					
				PTSD^a^^,^^b^	Agoraphobia^c^	PD^c^^d^	GAD^c^^e^	SAD^c^^,^^f^	MDD^c^^,^^g^
P1	60s	Accident in adulthood	3	(x)	x	N/A^h^	N/A	N/A	N/A
P2	40s	Accident in adulthood	8	x	x	x	x	N/A	N/A
P3	50s	Accident in childhood	6	(x)	N/A	N/A	N/A	x	N/A
P4	30s	Physical violence in adulthood	5	N/A	N/A	N/A	N/A	x	x
P5	50s	Physical and sexual violence in childhood	7	N/A	N/A	x	N/A	N/A	N/A
P7	30s	Physical and sexual violence in childhood^i^	7	x	N/A	N/A	N/A	N/A	N/A
P9	30s	Physical and sexual violence in childhood	3	x	N/A	N/A	x	x	x
P10	30s	Physical and sexual violence in childhood	3	(x)	N/A	N/A	N/A	x	N/A

a Assessed with Clinician-Administered PTSD Scale for DSM-5.

b PTSD: posttraumatic stress disorder. Subthreshold diagnoses are marked by (x).

c Assessed with MINI,

d PD: panic disorder.

e GAD: Generalized Anxiety Disorder Scale.

f SAD: social anxiety disorder.

g MDD: major depressive disorder.

h N/A: not applicable.

i One of P7’s memories was from an accident in adulthood.

### Study Design

This study is a case series that used a single symptom approach in which one intrusive memory was targeted at a time in different sessions [22,23,26]. Participants distinguished between different intrusive memories with a brief description. For example, if a participant had four different intrusive memories, the labels for each might be as follows: (1) angry face, (2) door opens, (3) clenched fist, and (4) blood (*note*: these examples are fictitious to protect anonymity). Participants then monitored the occurrence of each intrusive memory over time. The study design was the same as in our previous studies [22,23] and is described as a *repeated AB design* in which the length of the baseline (“A” preintervention, monitoring only) and intervention phases (“B”) varied for each specific memory, depending on which intervention session each memory was targeted in. Therefore, the baseline phase for each individual memory was used as a control period to compare their numbers before and after being targeted with the intervention.

Participants monitored the number of each intrusive memory in a daily diary for 1 week during the baseline phase, over a 6-week period maximum during the intervention phase, and then for 1 week during 1-month and 3-month follow-ups. Intrusive memories were targeted one at a time in up to 6 intervention sessions delivered remotely through a secure video platform. Participants could self-administer the intervention after the first intervention session if they chose to for the memories already targeted in session. The number of AB repetitions varied across participants: P1 received 3 repetitions, P2 received 4 repetitions, P3 received 2 repetitions, P4 received 3 repetitions, P5 received 2 repetitions, P7 received 6 repetitions, and P9 and P10 received 3 repetitions of AB design.

The primary outcome was the change in number of intrusive memories per week from baseline to the intervention phase and to the longer-term follow-ups (1 and 3 months). Participants also completed self-report measures for posttraumatic stress, depression and anxiety symptoms, and functional impairment (secondary outcomes) at baseline, the last intervention session, and the 1-month and 3-month follow-ups.

### Procedure

#### Overview

All sessions were delivered remotely through Kara Connect, a General Data Protection Regulation–compliant online platform allowing videoconferencing, certified by the Icelandic Directorate of Health. All data were recorded on a laptop computer using the REDCap (Research Electronic Data Capture) database, an encrypted, electronic software stored on secure servers [32]. The intervention was delivered by clinical psychologists (KT and JPH), who are experts in trauma-focused cognitive behavior therapy, 1 master-level student in clinical psychology, and 2 bachelor’s degree graduates.

#### Training of Researchers in Intervention Delivery

All people delivering the intervention underwent training, feedback, and continued clinical supervision from researchers or clinical psychologists (EAH and MK, who are experts in this intervention) to promote adequate intervention delivery and protocol adherence. Training for the clinical psychologists (n=2) included 2 in vivo workshops, the first for 3 days and then again approximately 6 months later for 2 days delivered by psychologists with expertise in developing and delivering the intervention in other settings [22,23]. The students (master-level student and the bachelor’s degree graduate [n=3]) participated in training workshops remotely via online video sessions. The workshops included topics such as theoretical background, practical aspects (including how to explain and use the primary outcome measure), and role-playing with trainers, with individual feedback provided until adequate performance was reached. Furthermore, all persons delivering the intervention received ongoing clinical supervision and support after each session via telephone from a clinical supervisor and in weekly group supervision meetings (EAH, MK, and ASB).

#### Baseline Session

Consistent with our procedure in previous studies [22,23], the researchers gave a brief description of intrusive memories in the baseline session. Participants then identified each of their different intrusive memories by giving a short verbal account of their visual content, using only a few words. Participants then labeled each intrusive memory with a symbol (eg, first memory labeled “A,” second memory “B,” etc) and then were instructed on how to monitor their occurrence in a daily diary (primary outcome measure). Participants indicated the frequency of each memory with the corresponding symbol in a specific time frame of that day. Each diary included 7 days and each day 4 time periods (see the “Measures” section); the baseline phase was 1 week in duration. Participants also completed baseline questionnaires in the baseline session (see the “Secondary Outcomes” section).

#### Intervention Sessions

In each intervention session (6 sessions maximum; intervention phase 6 weeks maximum) the participants selected 1 memory at a time to target that week and completed the intervention procedure guided by the researcher. The memory reminder here was the same one that was used in the study by Thorarinsdottir et al [22,23] where the participants were asked to briefly bring the visual content to mind without it becoming emotionally overwhelming. Participants were then taught how to use mental rotation and practiced before they played the Tetris computer game while using mental rotation for 25 minutes (ie, visualizing where to place and rotate game pieces before being played; for further information, see Holmes et al, Chapter 11 [17]). Participants played Tetris online on their computer (with marathon mode, selecting in the menu “ghost piece off” and sound set to 0%). Participants used share screen mode on Kara Connect (videoconferencing) so that the researcher could watch the participant playing and give feedback on using mental rotation throughout the duration of gameplay. Between sessions, the participants were invited to play Tetris at home in the same way as in sessions (ie, playing for 25 minutes using mental rotation), using either their smartphone or the computer, for already targeted intrusive memories, when an intrusion came to mind involuntarily. In the last intervention session, participants completed postintervention measures.

#### Follow-Up

At the 1-month and 3-month follow-ups, participants monitored the occurrence of intrusive memories in a daily diary for 1 week. They also completed follow-up measures that included the same questionnaires as completed in the baseline session and last intervention session.

### Measures

#### Eligibility Assessments (Part of the SAGA Cohort Substudy)

The CAPS-5 is a 30-item semistructured interview that assesses PTSD diagnosis and symptom severity in the past month, according to the PTSD criteria in the *DSM* [31]. Each item is scored on a 5-point Likert scale (0 = mild or subthreshold; 4 = extreme or incapacitating) with a symptom rating of 2 (ie, moderate) as a threshold for a possible diagnosis. The CAPS-5 has excellent internal consistency (Cronbach α=0.88), test-retest reliability (0.83), and good convergent validity (0.83) [33].

The MINI is a short, structured interview designed to assess axis I psychiatric disorders according to the *Diagnostic and Statistical Manual of Mental Disorders, Fourth Edition*. The interview has been shown to have good sensitivity and specificity for most diagnoses as well as good interrater reliability (κ=0.88‐1.0) and test-retest reliability (κ=0.76‐0.93) [34,35].

#### Primary Outcome Measure (Intrusive Memory Diary)

The intrusive memory diary was adapted from previous studies [18,20,36]. The number of intrusive memories was recorded in a daily pen-and-paper diary of 4 time points per day (morning, afternoon, evening, and night) for each week. The diary included a definition of intrusive memories of trauma as being mental images (pictures or a film clip in the mind’s eye) that occur involuntarily and cause distress. The participants are instructed not to record voluntary thoughts about the trauma or verbal thoughts without imagery. The participants monitored the occurrence of their intrusive memories daily for 1 week (baseline phase); for 6 weeks while intervention sessions were administered; and again, for 1 week at 1-month and 3-month follow-ups. Participants noted which intrusions came up, allowing us to investigate the change in frequency of each individual intrusive memory. The primary outcome was the number of intrusive memories from baseline phase to the intervention phase and again to 1- and 3-month follow-ups.

#### Secondary Outcome Measures

PTSD symptoms were assessed with the PCL-5, a brief 20-item self-report scale that assesses the severity of PTSD symptoms in the past month according to the *DSM-5* criteria for PTSD [37]. Symptoms are rated on a 4-point Likert scale (0 = not at all; 4 = extremely). The scale has been shown to have good internal consistency and test-retest reliability as well as good convergent and discriminant validity [37]. The Icelandic translation of the PCL-5 also has excellent internal consistency (α=.95) in the SAGA cohort study. A score of 33 on the PCL-5 is considered likely to detect PTSD cases according to *DSM-5,* and a score of 24 or below posttreatment is considered clinically significant [38].

Depressive symptoms were assessed with the Patient Health Questionnaire-9 (PHQ-9), a 9-item self-report measure of depression symptoms in the past 2 weeks, according to the *DSM-5* criteria [39]. Each item is rated on a 4-point Likert scale (0 = not at all; 3 = nearly every day). The PHQ-9 has good internal reliability (α = ranging from .86 to .89) and good test-retest reliability (*r*=0.84 [39]). The Icelandic version had good internal consistency in the SAGA cohort study (α=.89). A 5-point change on the PHQ-9 from pre- to posttreatment is considered clinically significant [40].

Anxiety symptoms were assessed with the Generalized Anxiety Disorder Scale (GAD-7), a brief self-report questionnaire that screens for symptoms of GAD and their severity in the past 2 weeks. Each item is scored on a 4-point Likert scale (0 = not at all; 3 = nearly every day). The GAD-7 has good test-retest reliability (*r*=0.83) and good convergent validity [41]. The Icelandic version has excellent internal consistency in the SAGA cohort study (*α*=.90). A 4-point change on the GAD-7 from pre- to posttreatment is considered clinically significant [42].

Functional impairment was assessed with the Sheehan Disability Scale (SDS), a self-report questionnaire used to assess functional impairment in the past week across three domains: (1) work or school, (2) social, and (3) family life [43]. These domains are measured on an 11-point scale (0 = not at all; 10 = extremely), which was adjusted to assess functional impairment associated with intrusive memories. The SDS has good internal consistency (*α*=.89) and construct validity [43], as well as the Icelandic version having good internal consistency in clinical groups (*α*=.84[44]). A 3-point change on the SDS has been used as a measure of treatment response [45].

Self-guided adherence for the usage of the intervention in daily life was assessed with a question regarding how often Tetris was played after experiencing an intrusive memory (11-point scale; 0 = not at all; 10 = every time).

Feasibility and acceptability for using a gameplay intervention to reduce intrusive memories were assessed with 2 self-rated items: “Would you recommend playing Tetris to a friend?” and “Do you consider gameplay to be an acceptable way to reduce the daily frequency of intrusive memories?” (10-point scale, higher score indicating greater acceptability or feasibility). In addition, 2 open-ended questions were asked: “How did you feel about playing Tetris after you had an intrusive memory?” and “Did you find the intervention helpful? If yes, how?”

The impact of intrusive memories on concentration, sleep, and stress was assessed with 6 self-rated items regarding the past week: two items assessing difficulties with concentration in general and due to intrusive memories (11-point scale; higher scores indicating more difficulties), duration of disruption following an intrusive memory (<1 minute to >60 minutes), 2 items assessing sleep difficulties due to intrusive memories (11-point scale; higher scores indicating more sleep difficulties), and 1 item assessing how much intrusive memories affected stress levels (0 = not at all; 10 = affected very much).

Ratings of general impact of intrusive memories involved 2 items on an 11-point scale (0 = not at all; 10 = very distressing or vivid): one assessing distress caused by intrusive memories and the other assessing how vivid they were in the past week.

Intrusion diary adherence was assessed with 1 item: “How accurately did you fill out the diary?” (0 = not at all; 10 = very accurately).

The impact of intrusive memories on daily functioning was assessed with 2 questions; one was open-ended: “Have the intrusive memories affected your ability to function in your daily life in the past week? If yes, how?” and the other one was self-rated: “Have the intrusive memories affected your ability to function in your daily life?” (11-point scale, higher score indicating greater impact on functioning).

### Data Analysis

#### Change in the Total Number of Intrusive Memories

Number of intrusive memories of trauma was recorded by participants in a diary daily (morning, afternoon, evening, and night) during the baseline phase for 1 week and each week during the intervention phase (weeks 1‐6) and during 1 week at 1-month and 3-month follow-ups. The primary outcome was the change in the number of intrusive memories of trauma. The time frame was baseline week, to the intervention phase (weeks 1‐6), and to follow-up (1 month and 3 months). In practice, due to scheduling reasons, in some instances, the baseline phase was longer than 1 week, and as anticipated, the number of intervention weeks varied. Therefore, since these periods had different time lengths, the mean number *per week* was calculated for comparability. Missing data were dealt with by excluding these time points from the calculations (see the “Results” section).

When examining change over time, percentage reduction in total intrusions per week was calculated from the baseline phase to the intervention phase to other time periods as following [1 − (mean number per week during intervention phase/mean number per week during baseline) × 100]. For example, for 1 participant, there were 19.87 intrusions per week in the baseline phase and 3.75 intrusions per week in the intervention phase. This was calculated as [1 − (3.75/19.87)] × 100=81.09% reduction in the intervention phase compared with baseline.

#### Change in the Number of Targeted Intrusive Memories Relative to Nontargeted Memories

Next, we examined the data per intrusive memory. Specifically, we examined the number per week of targeted memories in comparison with nontargeted ones. This was done by calculating the number in the same way as described [1 − (mean number per week during intervention phase/mean number per week during baseline) × 100] for targeted memories (ie, each of the targeted memories has different baseline and intervention periods). However, standard baseline and intervention periods were established for nontargeted memories (ie, same length of periods for all nontargeted memories) since they were not targeted with the intervention. The baseline for each nontargeted memory was 1 week (ie, before any memory was targeted) and the intervention phase was determined as the period from when any memory was targeted with the intervention.

#### Change in Other Symptoms and Functioning  

A descriptive approach was used to investigate whether clinically meaningful changes were observed in overall symptoms of posttraumatic stress, depression, anxiety, and functional impairment.

### Ethical Considerations

The study was approved by the National Bioethics Committee in Iceland (no. VSNb2017110046/03.01). All participants provided their written and informed consent. All data were anonymized. All sessions followed a written protocol. No adverse events related to the intervention were reported by participants. Participants were not provided with any form of compensation.

### Open Science Statement

The study was preregistered prior to study start on ClinicalTrials.gov (NCT04342416) on March 11, 2020. Anonymized summary-level data are reported in this manuscript. Study materials may be made available upon reasonable request with an appropriate material transfer agreement with Uppsala University (for the imagery competing task intervention and diary) or University of Iceland (all other measures). We note that delivery of this intervention at present requires the prerequisite training and supervision by psychologists with experience of developing it (see “Procedure” and “ Training of Researchers in Intervention Delivery” sections).

## Results

### Overview

**Figure 1.**
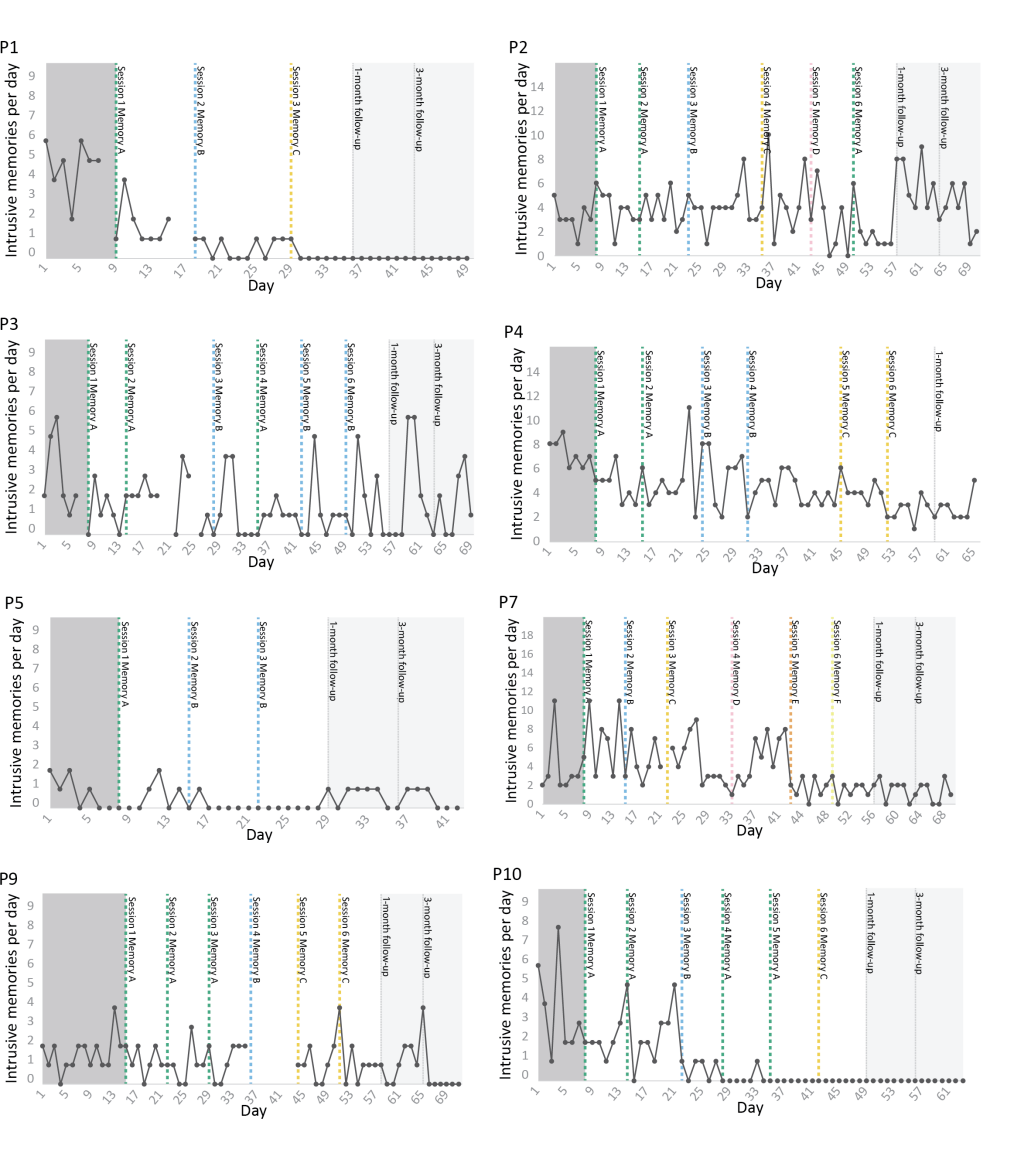
Total number of intrusive memories per day for each participant across phases; baseline, intervention, and follow-ups. Graphs for visual inspection of primary outcome data for each participant (on the *y*-axis as total number of intrusive memories per day, that is, for all distinct memories combined). Days since study start are shown on the *x*-axis, which includes baseline (gray), intervention (white), and follow-up periods (light gray). Dashed vertical lines show when each intervention session was administered, and which specific traumatic memory was targeted. Memories are labeled in order of when they were targeted (eg, ‘Memory A’ was targeted in the first intervention session). Dotted vertical lines show the 1-month and 3-month follow-up periods. Gaps in the time series represent missing data.

**Figure 2.**
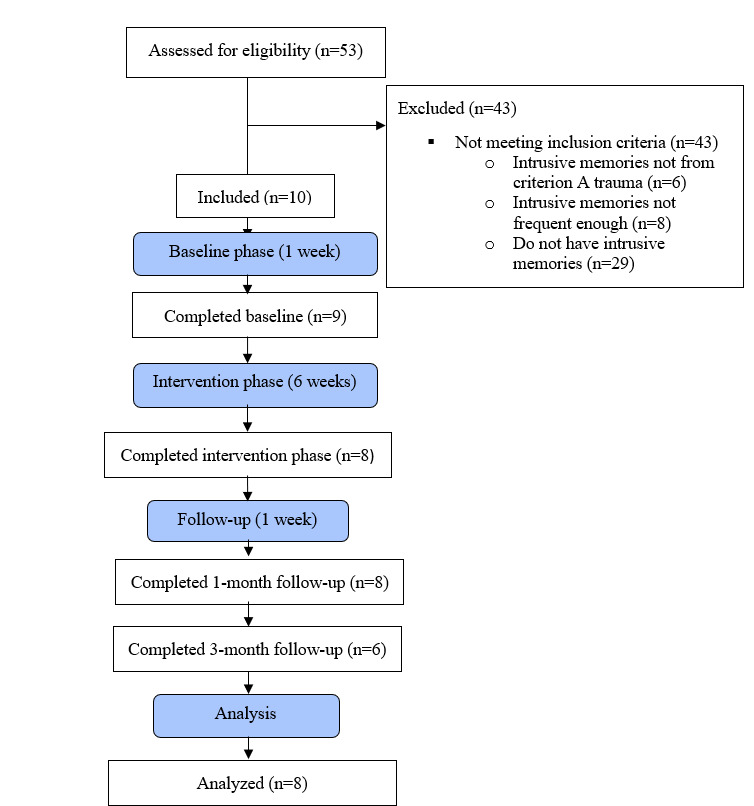
Adapted CONSORT (Consolidated Standards of Reporting Trials) flow diagram for the study.

Participants had varying numbers of intrusive memories at baseline (see Table 1 and Figure 1 for an overview). P1 monitored the frequency well, but data were missing for day 8 during the baseline and for days 16‐17 during the intervention phase. P2 monitored the frequency accurately with no missing data. P3 monitored the frequency quite accurately, but data were missing for day 7 during the baseline and for days 20‐21 and day 25 during the intervention phase. P4 monitored the frequency accurately with no missing data. P5 accurately monitored the frequency of her intrusive memories with no missing diary data. P7’s diary data were missing for day 22 during the intervention phase. P9 had missing diary data for 8 days during the intervention phase (days 36‐43) and P10 had no missing diary data. No attempts were made to retrieve missing diary data, and 97.5% of diary data were completed for all 8 participants (Figure 2).

### Primary Outcome

#### Change in the Total Number of Intrusive Memories

The total frequency per week for all intrusive memories during each phase for each participant (baseline, intervention, 1-month, and 3-month follow-ups) can be seen in Figure 1. It should be noted that each figure shows the frequency of intrusive memories over the whole week *after* the baseline session (leading up to the first intervention session), the frequency of intrusive memories the week *after* the first intervention session, and so on. Visual inspection for P1 shows that after the first intervention session, the total frequency of intrusions was reduced. The frequency continued to drop during the intervention phase and was zero at the follow-up periods. Visual inspection for P2 shows fluctuations in frequency over the intervention phase and an increase in frequency at follow-up. Visual inspection for P3 shows a noticeable drop in frequency after the first intervention session, followed by an increase after the second session. However, the frequency goes down in the third session and increases slightly over the rest of the intervention phase. The frequency increases at 1-month follow-up and then decreases again at 3-month follow-up. For P4, visual inspection shows the frequency of intrusions reducing after the first intervention session and remaining stable with some fluctuations over the intervention phase. The reduction is maintained at a 1-month follow-up. Visual inspection for P5 shows the frequency going down after the second intervention session and continuing to reduce over the intervention phase, although that reduction is not maintained during follow-up. For P7, there are fluctuations in the number of intrusive memories until after the fifth intervention session, where there is a clear drop in the frequency that is maintained through the follow-up periods. Visual inspection for P9 shows fluctuations in the number of intrusive memories from baseline throughout the intervention period, but the frequency is reduced at the 3-month follow-up period. For P10, the frequency seems reduced after the first intervention session and continues to drop after the third intervention session; the number of intrusive memories is zero after the fifth intervention session and remains so for the follow-up periods.

The mean number of targeted and nontargeted intrusions per week at the baseline phase, intervention phase, and at 1-month and 3-month follow-ups is shown in Table 2. Overall, the number of intrusive memories reduced for all participants from the baseline phase to the intervention, although the reduction was minimal for 2 participants. The range of percentage reduction from baseline to postintervention varied from 6.3% to 93.3%. For 5 out of 8 participants, the percentage reduction was larger at 1-month follow-up than postintervention from baseline, ranging from 27% to 100%. For 6 out of 8 participants, the percentage reduction was larger at 3-month follow-up than during the intervention phase compared with baseline, ranging from 58.3% to 100%. For P2, the number of intrusive memories was roughly the same at 3-month follow-up as at baseline (26 intrusive memories per week). The number of intrusive memories for P5 was higher (3 memories per week) at the 3-month follow-up than during the intervention phase (0.5 memories per week).

**Table 2.** Mean number of intrusive memories and percentage reduction per week across all phases; baseline, intervention, and at 1-month and 3-month follow-ups and total targeted and nontargeted intrusions for each participant.

Intrusions	Baseline (A)	Intervention (B)	Reduction (%)	1-month follow-up	Reduction (%)	3-month follow-up	Reduction (%)
Participant 1							
Total	28.6	2.5	91.40	0	100	0	100
Total targeted	28.6	2.5	—^a^	0	—	0	—
Total nontargeted	—	—	—	—	—	—	—
Participant 2							
Total	26.2	23.2	11.30	44	–68.10	26	—
Total targeted	21.2	14.7	—	21	—	20	—
Total nontargeted	5	8.6	—	23	—	6	—
Participant 3							
Total	17.1	10.1	41	15	12.30	10	41.50
Total targeted	13.6	8.5	—	2	—	10	—
Total nontargeted	3.5	1.6	—	13	—	0	—
Participant 4							
Total	50.3	28.9	42.50	19	62.20	—	—
Total targeted	47.3	27.8	—	19	—	—	—
Total nontargeted	3	1.1	—	0	—	—	—
Participant 5							
Total	7.5	0.5	93.30	5	33.30	3	60
Total targeted	6.5	0.5	—	3	—	3	—
Total nontargeted	1	0	—	2	—	0	—
Participant 7							
Total	32	24.1	24.80	11	65.70	9	71.90
Total targeted	27	21.3	—	11	—	8	—
Total nontargeted	5	2.8	—	0	—	1	—
Participant 9							
Total	9.6	9	6.30	7	27.10	4	58.30
Total targeted	9.6	9	—	7	—	4	—
Total nontargeted	—	—	—	—	—	—	—
Participant 10							
Total	19.9	3.8	81.10	0	100	0	100
Total targeted	19.9	3.8	—	0	—	0	—
Total nontargeted	—	—	—	—	—	—	—

a Not applicable.

#### Change in the Number of Targeted Intrusive Memories Relative to Nontargeted Memories

The number of targeted and nontargeted intrusions per week at baseline, postintervention, and at 1-month and 3-month follow-ups is displayed in Table 2. The mean number of individual targeted memories was 8.95 per week in the baseline phase and reduced to 6.11 per week over the intervention phase. The mean number of individual nontargeted memories was low at 2.74 per week in the baseline phase and reduced to 2.67 memories per week in the intervention phase. The number of individual targeted memories continued to reduce at the 1-month follow-up week to 2.33, and the number of nontargeted memories reduced to 1.80 at the 1-month follow-up. At the 3-month follow-up week, the mean number of individual targeted memories was further reduced to 1.67, and the mean of individual nontargeted memories was reduced to 0.33.

### Secondary Outcomes

#### Ratings of Adherence and General Impact of Intrusive Memories

The participants filled out the intrusion diaries quite accurately throughout the study (mean rating was 8.7/10, SD 1.16). The participants rated their intrusions in general as becoming less vivid and distressing over the intervention and follow-up phase. Seven out of the 8 participants self-administered the intervention at home during the intervention phase.

#### Feasibility and Acceptability for Using a Smartphone Gameplay Intervention

All participants rated the intervention to be an acceptable way to reduce the frequency of intrusive memories (mean 9.5, SD 0.8).

#### Self-Report Measures on PTSD, Depressive and Anxiety Symptoms, and General Functioning

The participants filled out questionnaires for PTSD, depressive and anxiety symptoms, and general functioning at baseline, after the last intervention session, and at follow-ups (Table 3). For the participant group, there was a reduction in PTSD symptoms for participants from a score of 47.6 at baseline measures on the PCL-5 to a score of 32.8 postintervention.

**Table 3.** Self-report measures for secondary outcomes and impact of intrusive memories on concentration, sleep, stress, and daily functioning mean for all participants.

Item	Baseline interview, mean (SD)	Postintervention, mean (SD)	1-month follow-up, mean (SD)	3-month follow-up, mean (SD)
PCL-5^a^	47.6 (15.2)	32.8 (16.5)	27.9 (19.4)	27.0 (17.9)
PHQ-9^b^	12.9 (8.0)	9.9 (6.7)	10.4 (7.1)	10.3 (7.4)
GAD-7^c^	10.8 (4.5)	6.6 (4.8)	6.4 (4.8)	7.7 (5.4)
SDS^d^	18.3 (8.2)	10.4 (5.7)	9.6 (8.0)	7.4 (7.0)
Concentration^e^	5.9 (1.7)	2.5 (2.2)	2.9 (2.2)	2.6 (2.1)
General concentration^f^	7.0 (1.7)	4.4 (2.9)	4.5 (3.4)	4.4 (3.2)
Duration of disruption^g^	2.3 (1.2)	1.8 (0.7)	1.4 (0.7)	0.7 (0.5)
Sleep^h^	4.8 (2.9)	1.5 (2.6)	1.9 (2.8)	1.3 (2.2)
Nightmares^i^	2.8 (3.6)	1.9 (2.9)	1.3 (2.6)	1.3 (2.4)
Stress^j^	5.8 (2.4)	3.4 (2.5)	2.8 (2.3)	2.4 (1.9)
Daily functioning^k^	4.4 (3.2)	2.0 (1.9)	2.3 (1.9)	2.1 (1.8)

a Posttraumatic Stress Disorder Checklist; scores ranging from 0 to 80.

b Patient Health Questionnaire-9; scores ranging from 0 to 27.

c Generalized Anxiety Disorder Scale; scores ranging from 0 to 21.

d Sheehan Disability Scale; scores ranging from 0 (unimpaired) to 30 (highly impaired).

e In the past week, how much did your intrusive memories disrupt your concentration? 0=not at all disruptive; 10=extremely disruptive.

f In the past week, how much difficulty did you have concentrating generally? 0=no difficulty concentrating; 10=extremely difficult to concentrate.

g When you had an intrusive memory, how long did it disrupt your concentration (in minutes) in the past week? 0 (<1 minute); 5 (>60 minutes).

h Did your intrusive memories interfere with sleep during the night in the past week? 0=not at all; 10=interfered very much.

i Did you experience any nightmares that interfered with your sleep during the night in the past week? 0=did not experience any nightmares; 10=experienced a lot of nightmares.

j In the past week, did your intrusive memories affect how stressed you felt? 0=not at all; 10=affected very much.

k Have the intrusive memories affected your ability to function in your daily life? 0=not at all; 10=affected very much.

The scores were further reduced in the 1-month follow-up to 27.9 and to 27.0 at the 3-month follow-up. Depressive symptoms were reduced from 12.9 at baseline to 9.9 postintervention, although slightly elevated at the follow-up phases (10.4 at 1-month follow-up and 10.3 at 3-month follow-up). Anxiety symptoms reduced from 10.8 at baseline to 6.6 postintervention and remained stable at 6.4 at the 1-month follow-up but were slightly increased to 7.7 at the 3-month follow-up. Functional impairment was reduced in a clinically significant way for participants from 18.3 at baseline to 10.4 postintervention. At follow-ups, the reduction in impairment in functioning related to the intrusions continued to drop to 9.6 at 1-month follow-up and to 7.4 at the 3-month follow-up (Table 3).

#### Impact of Intrusive Memories on Concentration, Sleep, Stress, and Daily Functioning

Table 3 also shows ratings of the impact of intrusions on concentration, stress, sleep, and daily functioning during all phases. Concentration difficulties related to the intrusions were lowered from baseline to postintervention and were maintained at follow-up. Ratings on concentration in general showed a similar pattern. The effect on estimated duration of disruption following an intrusion was reduced from 2.3 at baseline to 1.8 postintervention, to 1.4 at 1-month follow-up, and to 0.7 at 3-month follow-up. The effect the intrusive memories had on sleep reduced from 4.8 at baseline to 1.5 postintervention. These improvements in sleep were maintained at follow-up. Participants’ nightmares reduced from baseline to postintervention and to follow-up phases. Participants reported less stress related to the intrusive memories postintervention (3.4) compared with baseline (5.8), which continued to reduce to 2.8 at 1-month follow-up and to 2.4 at 3-month follow-up. The impact that intrusive memories had on the ability to function in daily life was 4.4 at baseline and was reduced to 2.0 postintervention; this reduction was maintained at follow-up phases.

## Discussion

### Principal Findings

In this case series, we built on our previous case study (n=1) [22] and case series (n=3) [23] and further investigated the effects of a novel intervention to reduce intrusive memories of past trauma. The total number of intrusive memories (primary outcome) of trauma occurring years earlier was reduced from baseline to the intervention phase among 6 out of 8 participants (25%-93% reduction). For 5 out of 8 participants, the number of intrusive memories continued to reduce to follow-up phases. Two participants reported zero intrusions at follow-up. In general, the frequency of targeted intrusions reduced more than that of nontargeted intrusions.

The overall trend for secondary outcomes was that symptoms of PTSD, depression, and anxiety reduced significantly for most participants from baseline to postintervention, and symptoms continued to reduce to follow-ups for most of them. Symptoms were reduced in a clinically significant way on the measure of anxiety but not on measures of PTSD and depression. Functional impairment was improved for participants and continued to improve at follow-up phases. Participants reported less stress related to intrusive memories and their sleep was improved. The time intrusive memories interrupted concentration was lessened from approximately 30-minute disruption at baseline to approximately 5‐10 minutes postintervention and continued to reduce to about 1‐ to 5-minute disruption at the 3-month follow-up. The effect intrusive memories had on participants’ ability to function in daily life was also improved from baseline to postintervention, and this improvement was maintained at follow-up.

Findings for the primary outcome are similar to the study by Kessler et al [26] (reduction of 64% overall) and our previous case study [22] and case series [23] where the reduction in the number of intrusive memories ranged between 38% and 56%. These findings also follow the same trend as the results found in the study by Kessler et al [26] in which targeted intrusions reduced by 64% and nontargeted intrusions reduced by 11%. However, it should be noted that here the number of nontargeted intrusions was very low at baseline. Overall, the longer-term follow-up results on symptoms reduction over time are in accordance with our previous case study [22] and case series [23]. These results suggest the potential longer-term effects on intrusions, which could be due to the simplicity of self-administered use of the intervention, giving participants the chance to use it independently if needed once they have been taught how to use it.

Future research should explore whether self-guided use that extends to all intrusive memories instead of just targeted ones would result in greater reductions in intrusive memories. It is not clear why 2 participants did not experience clear treatment gains. More research is needed with larger samples in order to determine predictors of treatment response and hopefully to adjust the intervention if needed to different groups.

Follow-up results may suggest that clinically targeting core symptoms such as intrusive memories in PTSD can have effects on other symptoms in the symptom network [46,47]. It should be highlighted that in this study, all sessions were carried out remotely, and all participants found the intervention to be an acceptable way to reduce intrusive memories and that they would certainly recommend it to a friend, similar to previous research [22,23]. Delivery of the intervention in person does not seem to be essential, and remote delivery appears acceptable and effective.

 This intervention developed to target one symptom of PTSD does not include some of the barriers that current PTSD treatment entails, including patients’ reluctance to recall the traumatic event in detail, high cost, and geographical barriers [11]. In the current case series, the intervention was delivered completely remotely, removing geographical constraints, thus being able to reach people regardless of their location or situation (eg, in quarantine during the COVID-19 pandemic [28]). Results to date are promising. However, future research should move toward larger-scale testing and comparing the remotely delivered intervention with a credible control task.

Critically, taking one step further toward simplicity, the intervention was delivered to 4 participants (half of the participants) by a master-level student and 2 bachelor degree graduates, that is, not mental health professionals, after receiving training in the intervention. This did not affect the results, which indicates that nonspecialists in trauma therapy could successfully deliver the intervention after appropriate training. Treating people posttrauma without the need for a mental health specialist (as only some of our researchers were clinically qualified) is a novel contribution of this research study and one that, if tested further in a randomized controlled trial (RCT), could help improve the scalability of treatment delivery. It is important to test this intervention with case series involving other groups, such as men and more diverse populations, before moving on to an RCT, which could help improve the scalability of treatment delivery. This could mean after RCT validation that the intervention could benefit other groups after trauma, for example, regarding ethnicity and gender.

### Limitations

This case series included participants of 1 group (Icelandic women), as well as including a small sample size, therefore limiting the generalizability of results. Furthermore, we cannot estimate the effects of common factors of treatment as we did not include a credible control task.

### Conclusions

When delivered remotely, and by nonmental health professionals, this imagery competing task intervention (including a brief memory reminder cue + Tetris gameplay with mental rotation) showed promising results in reducing the frequency of intrusive memories for women with trauma that could be from many years back. Results confirm and extend previous studies involving older trauma memories. Targeting intrusive memories of trauma appeared to impact PTSD, depression, and anxiety symptoms (secondary outcomes), which may suggest that such targeted efforts can have effects on other symptoms in the symptom network. Delivering the intervention remotely was feasible and acceptable to all participants. Participants opted for varying numbers of intervention sessions (ranging from 3 to 6 sessions). One study has found that similar gains could be achieved with fewer sessions. However, more research is needed [48]. Furthermore, it should be explored how psychoeducation could be added to the intervention package in the form of simple videos explaining how intrusive memories are formed and how the intervention works and why it can be effective—further moving the intervention delivery toward self-guided delivery. The next steps in developing the intervention and assessing its effectiveness could include fewer sessions, less researcher support, and having nonmental health professionals deliver the intervention. Although the results are promising, these findings are preliminary since the efficacy of the intervention needs to be validated with a larger-scale RCT comparing the intervention with a credible control task.

## Abbreviations

CAPS-5: Clinician-Administered PTSD Scale for DSM-5
CONSORT: Consolidated Standards of Reporting Trials
*DSM-5*: *Diagnostic and Statistical Manual of Mental Disorders, Fifth Edition*
GAD-7: Generalized Anxiety Disorder Scale
MINI: Mini International Neuropsychiatric Interview
PCL-5: Posttraumatic Stress Disorder Checklist
PHQ-9: Patient Health Questionnaire-9
PTSD: posttraumatic stress disorder
RCT: randomized controlled trial
REDCap: Research Electronic Data Capture
SAGA: Stress and Gene Analysis
SDS: Sheehan Disability Scale
